# Long-term exposure to air pollution and traffic noise and incidence of mental disorders: a large administrative cohort of adults

**DOI:** 10.1192/j.eurpsy.2023.1124

**Published:** 2023-07-19

**Authors:** A. Forastiere, F. Nobile, M. Stafoggia

**Affiliations:** Department of Epidemiology, Local Health Authority Rome 1, Rome, Italy

## Abstract

**Introduction:**

Air pollution is related to a global increase in mortality and morbidity. The literature on the adverse effects on mental disorders is still limited.

**Objectives:**

This study aims to investigate the associations between air pollutants and traffic noise with incidence of different categories of mental disorders and drug prescriptions in a large cohort administrative cohort.

**Methods:**

We enrolled 1,739,277 individuals 30+ years living in Rome at 2011 census, and followed them up until 31^st^ December 2019. We excluded subjects with prevalent mental disorders at baseline to evaluate the incidence of schizophrenia, bipolar, anxiety, personality and substance use disorders, as well as prescriptions of antipsychotics, antidepressants and anticonvulsants. We assigned annual average concentrations of fine particulate matter (PM_2.5_), nitrogen dioxide (NO_2_), Black Carbon (BC), ultrafine particles (UFPs) and noise exposure to baseline residential addresses. We applied Cox regression models with adjustment for individual and area-level covariates.

**Results:**

This study identified variable numbers of incident cases, from 1,280 cases for personality disorders to 200,549 for antidepressants. Each interquartile range increase in PM_2.5_ (1.13μg/m^3^) was associated with a hazard ratio (HR) of 1.07 (95% confidence interval: 1.017, 1.127) for schizophrenia spectrum disorder, 1.135 (1.086, 1.186) for depression, 1.097 (1.030, 1.168) for anxiety disorders and 1.112 (1.030-1.200) for substance use disorders. Positive associations were also detected for the other exposures and with the three categories of drug prescriptions. In two-exposure models, PM_2.5_, UFPs and noise remained associated with schizophrenia spectrum disorders, depression and antidepressant drugs use. The effects were higher in the age group 30-64 than in the 65+. Sensitivity analyses generally yielded similar results

**Conclusions:**

Long-term exposure to air pollutants and noise was associated with increased risks of schizophrenia spectrum disorders, depression and anxiety disorders. The associations with prescriptions of specific drugs increase the credibility of the results.

**Disclosure of Interest:**

None Declared

